# Risk stratification of patients with acute respiratory distress syndrome complicated with sepsis using lactate trajectories

**DOI:** 10.1186/s12890-022-02132-6

**Published:** 2022-09-07

**Authors:** Haoyue Zhang, Ziping Li, Weiqiang Zheng, Linlin Zhang, Tianqi Yang, Keliang Xie, Yonghao Yu

**Affiliations:** 1 The Graduate School, Tianjin Medical University, Tianjin, China; 2 Department of Anesthesiology, Tianjin Medical University General Hospital, Tianjin, China; 3 Department of Cardiology, Tianjin Medical University General Hospital, Tianjin, China; 4 Department of Critical Care Medicine, Tianjin Medical University General Hospital, Tianjin, China

**Keywords:** Lactate, Acute respiratory distress syndrome, Sepsis, Group-based trajectory modeling, Multivariable fractional polynomial interaction

## Abstract

**Background:**

No consensus has been reached on an optimal blood lactate evaluation system although several approaches have been reported in the literature in recent years. A group-based trajectory modeling (GBTM) method could better stratify patients with acute respiratory distress syndrome (ARDS) complicated with sepsis in the intensive care unit (ICU).

**Patients and methods:**

760 patients from the comprehensive ICU of Tianjin Medical University General Hospital with ARDS complicated with sepsis were eligible for analysis. Serial serum lactate levels were measured within 48 h of admission. In addition to the GBTM lactate groups, the initial lactate, peak lactate level, the area under the curve of serial lactate (lactate AUC), and lactate clearance were also considered for comparison. The short- and long-term outcomes were the 30- and 90-day mortality, respectively.

**Results:**

Three lactate groups were identified based on GBTM, with group 3 exhibiting the worse short- [hazard ratio (HR) for 30-day mortality: 2.96, 95% confidence interval (CI) 1.79–4.87, *P* < 0.001] and long term (HR for 90-day mortality: 3.49, 95% CI 2.06–5.89, *P* < 0.001) outcomes followed by group 2 (HR for 30-day mortality: 2.05, 95% CI 1.48–2.84, *P* < 0.001 and HR for 90-day mortality: 1.99, 95% CI 1.48–2.67, *P* < 0.001). GBTM lactate groups exhibited significantly improved diagnostic performance of initial lactate + SOFA scores/APACHE II scores models. Based on the multivariable fractional polynomial interaction (MFPI) approach, GBTM lactate groups could better differentiate high-risk patients than the initial lactate groups in short- and long-term outcomes.

**Conclusions:**

To the best of our knowledge, this is the first report that GBTM-based serial blood lactate evaluations significantly improve the diagnostic capacity of traditional critical care evaluation systems and bring many advantages over previously documented lactate evaluation systems.

**Supplementary Information:**

The online version contains supplementary material available at 10.1186/s12890-022-02132-6.

## Background

Elevated lactate levels are common in the setting of sepsis and usually represent an imbalance between increased production in the presence or absence of tissue hypoxia and changes in clearance [1]. Given their well-established association with multiple organ dysfunction [2], blood lactate levels and their trend over time are increasingly recognized as reliable markers of illness severity and mortality [3–7]. Border and Weil et al. observed that lactate levels ≥ 4 mmol/L were associated with a 50% mortality in patients with shock during a study period of 36 h [8]. An increasing body of evidence suggests that lactate measurements represent a valuable and straightforward indicator for the early identification of severe sepsis and are associated with poor outcomes [9–13]. Interestingly, current evidence suggests that compared with initial lactate levels at presentation or other single measured lactate values, serial blood lactate level measurements can better characterize the dynamic process of lactate production and elimination in vivo [2]. Serial lactate levels can also help define the patient's trajectory and encourage a review of the therapeutic strategy [14]. Over the years, the prognostic value of serial measurements of multiple types of lactate levels or calculations from lactate levels has been investigated, for example, the duration of hyperlactatemia [15], the area under the curve of serial lactate measurement (lactate AUC) [16], and lactate clearance [17]. Importantly, decreased lactate levels were associated with improved outcomes in almost all critically ill patients [5]. Moreover, the later the lactate level is reduced, the worse the clinical prognosis is [18].

Despite the long-standing predictive power and the prominent place of lactate levels in early goal-directed therapy for critically ill patients, there is still a lack of a dynamic and flexible method to identify high-risk patients with hyperlactatemia. Given the lag in blood lactate concentration observed during testing, it remains unclear how lactate levels affect clinical management [19]. Notwithstanding that it is well-recognized that poor outcomes in ARDS patients are often attributed to sepsis/multiple organ failure rather than respiratory failure per se [20], few studies have hitherto focused on the predictive value of lactate for the prognosis of acute respiratory distress syndrome (ARDS) patients, along with the hypoxia process [1]. Indeed, a better understanding of the association between lactate and poor prognosis in ARDS patients complicated with sepsis upon admission is required.

We validated that lactate levels are elevated in septic patients with ARDS based on cohort data, including serial measurements of blood lactate over a period. A recently developed statistical method, also known as group-based trajectory modeling (GBTM), was harnessed to identify clusters of individuals exhibiting similar progression of a specific measurement over time and to identify the association between lactate trajectory within 48 h and short- and long-term outcomes of ARDS patients complicated with sepsis. Meanwhile, although several lactate evaluation systems have been documented, including initial serum lactate levels, we sought to validate that novel subtypes of lactate based on GBTM algorithm yield better diagnostic value.

## Methods

### Study design, patients

This was a single-center, retrospective observational study where patients discharged from the comprehensive intensive care unit of Tianjin Medical University General Hospital from January 2019 to March 2022 were enrolled. Patients with the following conditions were excluded: (1) patients discharged within 48 h of admission; (2) patients without admission arterial blood gas analysis measurements; (3) patients not diagnosed with ARDS and sepsis; and (4) patients with repeated admissions. The diagnosis of ARDS was made according to the criteria stated by the American-European Consensus Conference (AECC) on ARDS at admission [21]. In addition, we excluded patients whose clinical entities can present with PaO_2_/FiO_2_ < 300 mmHg without being ARDS (atelectasis, volume overload, and left heart failure). The diagnosis of sepsis during ICU stay is based on the consensus Sepsis-3 definitions at admission [9], which is the SOFA score of the patient within 24 h of admission. We routinely measure the plasma lactate concentration (via long-term arterial catheterization) by a COBAS B-123 POC system (Roche Diagnostics, Rotkreuz, Switzerland) every 6 h in all patients during ICU stay. Besides the initial lactate measurement, missing values were allowed for the other time points. The patients were followed up through outpatient visits, telephone calls or hospital readmission records. This study was approved by the Ethics Committee of Tianjin Medical University General Hospital (IRB2022-YX-041-01) and conducted in accordance with the ethical standards of the Declaration of Helsinki. The need for informed consent was waived due to the retrospective nature of our study. Reporting of the study followed the guidelines from the Strengthening the Reporting of Observational Studies in Epidemiology (STROBE) statement [22]. We declare that ARDS due to coronavirus disease 2019 (COVID-19) is not involved in patients eligible for inclusion.

### Study variables

The study variables used for analysis included patient demographics (age, sex), past medical history (hypertension, diabetes, coronary artery disease, stroke, renal failure, cancer), clinical characteristics upon admission [levels of systolic and diastolic blood pressure (SBP and DBP), heart rate, estimated glomerular filtration rate (eGFR), fluid intake per hour, fluid outtake per hour, red blood cell (RBC), white blood cell (WBC), platelet, hemoglobin, alanine aminotransferase (ALT), aspartate aminotransferase (AST), albumin, Glasgow Coma Scale (GCS) [23], Acute Physiology And Chronic Health Evaluation II (APACHE II) score [24] and Sequential Organ Failure Assessment (SOFA) score [25], PaO_2_/FiO_2_ classification, emergency surgical operation, invasive mechanical ventilation, and other inpatient treatment within 24 h of admission [antibiotics, sedative/analgesic, glucocorticoid, continuous renal replacement therapy (CRRT), low molecular weight heparin (LMWH), sodium bicarbonate, vasoactive medications, and blood component transfusion]. The eGFR was calculated according to the Chronic Kidney Disease Epidemiology Collaboration equation [26]. The definition of the above study variables is listed in Additional file 1: Table S1. Except for the APACHE II and SOFA scores, all the above parameters were included as covariables in the multivariate Cox proportional-hazards model. In addition, the initial lactate was also included during model adjustment.

In addition to the lactate groups based on the GBTM algorithm (GBTM lactate groups), the following lactate evaluation criteria were also considered in this study for comparison: (1) initial lactate level; (2) peak lactate level; (3) lactate AUC; (4) lactate clearance. The calculation of lactate AUC is shown in Additional file 1: Fig. S1.

For variables with missing values, we imputed the missing data using MissForest, a random forest imputation algorithm for missing data implemented in R (version 4.0.0) [27]. The missing rates of the study variables are shown in Additional file 1: Table S2.

### Study outcomes

The study outcomes of this study were the performance of GBTM lactate groups in the prediction of the short- (30-day mortality) and long-term (90-day mortality) outcomes.

### Statistical analysis

STATA 17.0 (StataCorp, College Station, TX) was used for statistical analysis. A two-tailed *P*-value < 0.05 was statistically significant. To capture the pattern changes of each blood lactate parameter, we used GBTM to identify distinct subgroups that shared a similar trajectory. Since the blood lactate levels were not normally distributed, the data were first logarithmically transformed. We fitted models with up to three latent classes and up to cubic polynomials. The optimal number of trajectory classes and trajectory shapes were selected based on the following criteria [28]: (1) a censored normal model for continuous tendency toward a parsimonious model, (2) the higher value of Bayesian information criterion (BIC) and Akaike information criterion (AIC), (3) each trajectory group had the odds of correct classification by weighted posterior proportions > 5, (4) each trajectory group had an average posterior probability of membership > 0.80 and (5) the expected number based on the sums of the posterior probabilities should be the same as in the proportion in each group based on the assignments for the maximum posterior probability.

To determine whether GBTM lactate groups could refine the diagnostic performance for short- and long-term outcomes, we compared the changes in AUC, net reclassification improvement (NRI) and integrated discrimination improvement (IDI) after incorporating the identified GBTM lactate groups, peak lactate value, lactate clearance and lactate AUC into the established models (initial lactate + SOFA score/APACHE II score).

The baseline characteristics between groups were reported as the means (± SD) for normally distributed data and medians with 25th and 75th percentiles for non-parametric data. Categorical variables were summarized using percentages. Differences between groups were compared using Fisher's exact test for data with a binomial distribution. The Mann–Whitney *U* test and unpaired *t*-test were used for group comparisons of continuous variables with skewed and normal distributions, respectively. The associations between GBTM lactate groups and short- and long-term risk were estimated using multivariable Cox proportional hazard analyses. Box-Whisker plots and 3D scatter plots were used to describe the GBTM lactate group characteristics by prior lactate evaluation systems.

Finally, the GBTM lactate groups and initial lactate groups were compared. The initial lactate groups were defined as group 1: ≤ 2 mmol/L; group 2: 2–4 mmol/L; and group 3: ≥ 4 mmol/L. We used the multivariable fractional polynomial interaction (MFPI) approach [29] to investigate potential interactions between group assignment (group 2 vs. 1 and group 3 vs. 1) and continuous variables such as age, eGFR, SOFA score, and APACHE II score for short- and long-term outcomes. All MFPI analyses were adjusted for covariates mentioned above. We decided to use only fractional polynomial degree 2 functions to analyze interactions. MFPI was used to estimate a fractional polynomial function for each group representing the prognostic effect of the continuous covariate of interest, optionally adjusting for other covariates. The covariables involved in adjustments are described in the section "2.2 Study Variables". A *P*-value < 0.05 was statistically significant for covariate selection. To quantify the magnitude of effects, we estimated hazard ratio (HR) with pointwise 95% confidence intervals (CI) as a continuous treatment effect. We adopted the 10th to the 90th quantile of distribution for the four continuous indicators.

## Results

### Patient characteristics

**Fig. 1:**
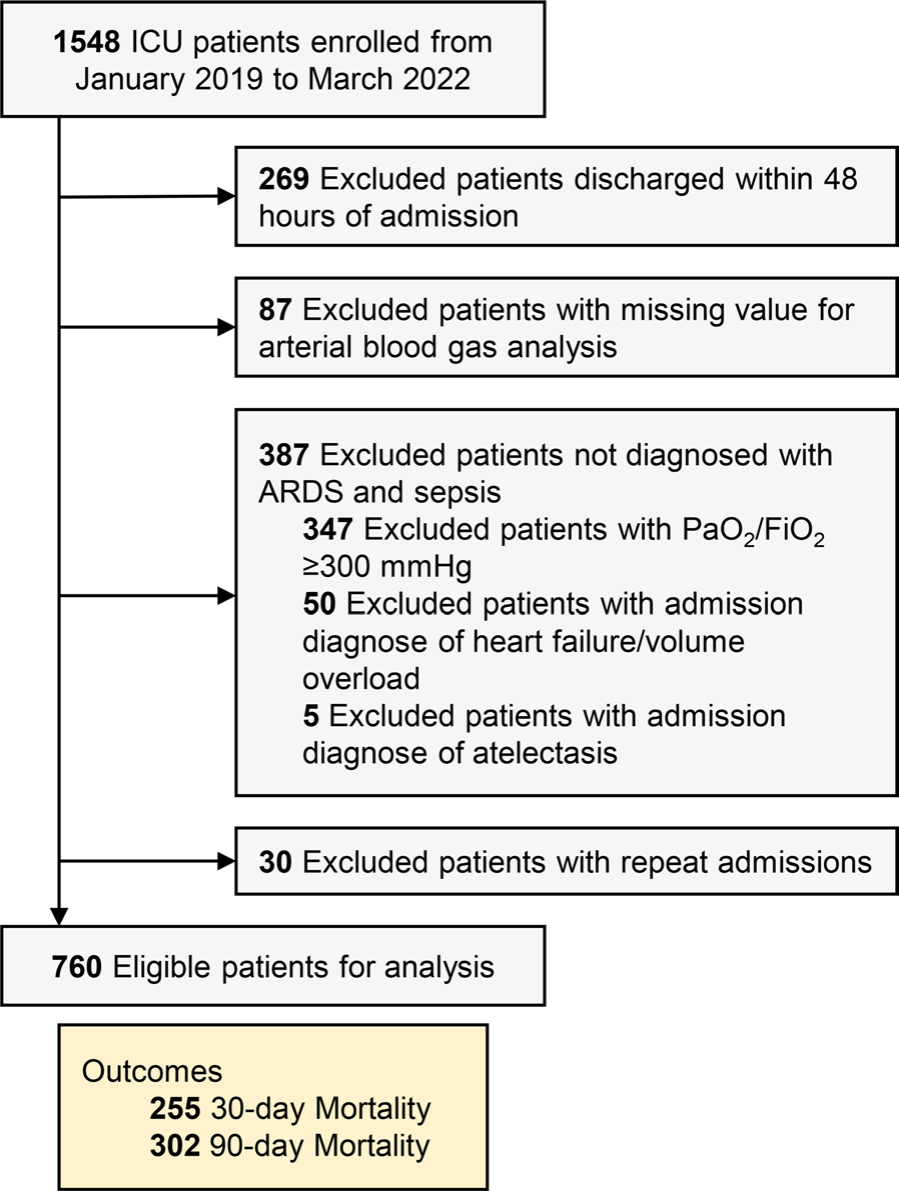
A schematic overview illustrating participant enrollment and the exclusion and inclusion criteria. ICU, intensive care unit

From January 2019 to March 2022, 1,548 patients were discharged from the comprehensive ICU of Tianjin Medical University General Hospital. As shown in Fig. 1, 760 patients were included in the final analysis. 255 and 302 patients experienced mortality at 30- and 90-day, respectively. There were no cases of loss to follow-up. The initial diagnosis of these eligible patients when admitted to ICU is shown in Additional file 1: Table S3. Comparisons of short- and long-term outcomes are shown in Additional file 1: Table S4. For both short- and long-term outcomes, lactate levels at all time points within 48 hours, peak lactate levels, and lactate AUC were significantly higher in the group with adverse events.

### Trajectories of serial blood lactate within 48 h after admission to the ICU based on the GBTM algorithm

**Fig. 2:**
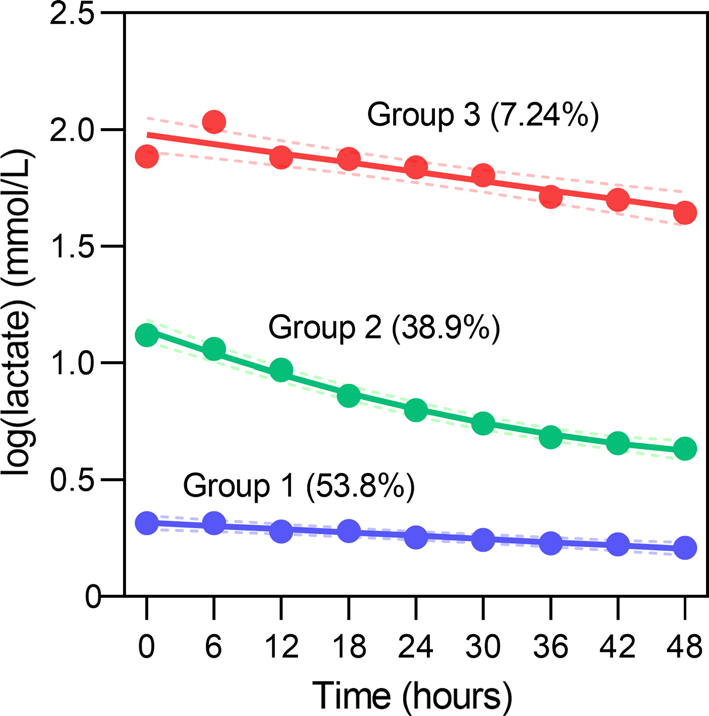
The blood lactate trajectories based on GBTM. The data were log-transformed before modeling. The dashed lines indicate 95% confidence intervals of each trajectory

As shown in Fig. 2, based on the GBTM algorithm, after considering parsimony and assessing the relative changes in model fitting at each step, we selected a model with three latent groups and linear, quadratic, and linear polynomials (see Additional file 1: Table S5 for parameters for models fitted with different numbers of latent groups and different degrees of polynomials). GBTM lactate groups 1, 2, and 3 accounted for 53.8% (n = 409), 38.9% (n = 296) and 7.24% (n = 55) of patients, respectively. The baseline characteristics of the groups are shown in Table 1. The blood pressure, platelet count, and albumin were significantly reduced. In contrast, heart rate, fluid intake per hour, WBC, ALT, AST, APACHE II score, and SOFA scores were significantly increased from group 1 to group 3 and required more treatment, including emergency surgical operation, invasive mechanical ventilation support, sedative/analgesic, glucocorticoid, CRRT, intravenous sodium bicarbonate, vasoactive medications, and blood component transfusion.

**Table 1:** Baseline characteristics

Characteristics	Total n = 760	GBTM lactate groups			*P* value
		Group 1 n = 409	Group 2 n = 296	Group 3 n = 55	
*Demographics*					
Age, year	62.2 ± 16.7	61.5 ± 17.9	63.2 ± 15.6	62.1 ± 13.4	0.400
Male, n (%)	464 (61.1)	258 (63.1)	178 (60.1)	28 (50.9)	0.200
*Previous history*					
Hypertension, n (%)	401 (52.8)	224 (54.8)	153 (51.7)	24 (43.6)	0.270
Diabetes, n (%)	235 (30.9)	141 (34.5)	82 (27.7)	12 (21.8)	0.054
CAD, n (%)	174 (22.9)	109 (26.7)	54 (18.2)	11 (20.0)	0.029
Stroke, n (%)	167 (22.0)	100 (24.4)	59 (19.9)	8 (14.5)	0.150
Renal failure, n (%)	76 (10.0)	53 (13.0)	19 (6.40)	4 (7.30)	0.014
Cancer, n (%)	95 (12.5)	34 (8.30)	49 (16.6)	12 (21.8)	< 0.001
*On-admission clinical characteristics*					
SBP, mmHg	137.3 ± 35.1	145.3 ± 34.2	130.7 ± 33.2	113.3 ± 33.9	< 0.001
DBP, mmHg	70.3 ± 19.1	74.6 ± 18.7	66.9 ± 18.1	57.5 ± 17.7	< 0.001
Heart rate, bpm	101.7 ± 24.1	97.9 ± 23.0	105.1 ± 25.2	110.7 ± 21.2	< 0.001
eGFR, mL/min/1.73m^2^	58.8 ± 41.7	60.8 ± 44.2	58.0 ± 38.7	47.7 ± 36.6	0.082
Fluid intake, mL/h	161.9 (113.3–243.9)	151.7 (106.8–226.9)	166.3 (116.3–230.5)	276.5 (168.8–441.6)	< 0.001
Fluid outtake, mL/h	136.0 (84–234.9)	141.5 (85.6–227.8)	126.3 (79.0–231.9)	145.5 (94.1–316.0)	0.200
RBC, 10^9^/L	3.36 (2.62–4.12)	3.40 (2.67–4.14)	3.35 (2.64–4.07)	3.04 (2.53–3.96)	0.210
WBC, 10^9^/L	10.8 (7.70–16.0)	10.4 (7.54–14.2)	11.7 (8.17–17.8)	12.3 (7.39–17.4)	0.016
Platelet, 10^9^/L	165.0 (93.0–227.0)	179.0 (117.0–240.0)	146.0 (73.0–210.5)	102.0 (37.0–170.0)	< 0.001
Hemoglobin, g/L	101.0 (80.0–124.5)	101.0 (80.0–125.0)	104.0 (80.0–123.5)	94.0 (74.0–125.0)	0.570
ALT, U/L	36.0 (22.0–77.0)	29.0 (19.0–56.0)	44.0 (23.0–90.0)	67.0 (28.0–224.0)	< 0.001
AST, U/L	53.0 (32.0–116.0)	43.0 (28.0–76.0)	66.0 (36.0–168.0)	133.0 (63.0–428.0)	< 0.001
Albumin, g/L	30.0 (26.0–34.0)	31.0 (28.0–35.0)	30.0 (26.0–33.0)	25.0 (22.0–30.0)	< 0.001
GCS Score	13 (7–15)	14 (8–15)	11 (5–15)	12 (4–15)	< 0.001
APACHE II Score	18 (13–24)	17 (12–22)	20 (16–26)	21 (15–31)	< 0.001
SOFA Score	11 (8–14)	10 (8–12)	12 (10–16)	17 (13–20)	< 0.001
*PaO*_*2*_*/FiO*_*2*_*, mmHg*					< 0.001
200–300	307 (40.4)	202 (49.4)	90 (30.4)	15 (27.3)	
100–200	323 (42.5)	171 (41.8)	123 (41.6)	29 (52.7)	
< 100	130 (17.1)	36 (8.80)	83 (28.0)	11 (20.4)	
Emergency surgical operation, n (%)					
	93 (12.2)	31 (7.60)	49 (16.6)	13 (23.6)	< 0.001
Invasive mechanical ventilation, n (%)					
	482 (63.4)	210 (50.0)	224 (75.7)	48 (87.3)	< 0.001
*Other in-hospital treatment within 24 h of admission*					
Antibiotics, n (%)	676 (88.9)	353 (86.0)	273 (92.2)	50 (90.9)	0.043
Sedative and analgesic, n (%)	472 (62.1)	238 (57.1)	194 (65.5)	40 (72.7)	0.036
Glucocorticoid, n (%)	353 (46.4)	137 (32.2)	182 (61.1)	35 (63.6)	< 0.001
CRRT, n (%)	260 (34.2)	130 (33.5)	100 (33.8)	30 (54.5)	0.005
LWMH, n (%)	101 (13.3)	65 (14.7)	33 (11.1)	1 (1.80)	0.003
Sodium bicarbonate, n (%)	358 (47.1)	162 (39.3)	158 (53.4)	38 (69.1)	< 0.001
Vasoactive medications, n (%)	245 (32.2)	81 (19.5)	122 (41.2)	42 (76.4)	< 0.001
Blood component transfusion, n (%)	274 (36.1)	119 (27.4)	117 (39.5)	38 (69.1)	< 0.001

ALT, alanine aminotransferase; APACHE II, Acute Physiology And Chronic Health Evaluation II; AST, aspartate aminotransferase; CRRT, continuous renal replacement therapy, DBP, diastolic blood pressure; eGFR, estimated glomerular filtration rate; GBTM, group-based trajectory modeling; GCS, Glasgow Coma Scale; LMWH, low molecular weight heparin; RBC, red blood cell; SBP, systolic blood pressure; SOFA, Sequential Organ Failure Assessment; WBC, white blood cell

As shown in Additional file 1: Fig. S2A, compared with group 1, groups 2 and 3 were characterized by significantly increased initial lactate, peak lactate level, lactate clearance, and lactate AUC (Additional file 1: Table S6 for details). A "funnel shape" 3D scatter plot was observed when three dimensions (initial lactate, peak lactate value, and lactate AUC) were considered (Additional file 1: Fig. S2B).

### Associations between GBTM lactate groups and short- and long-term outcomes

**Fig. 3:**
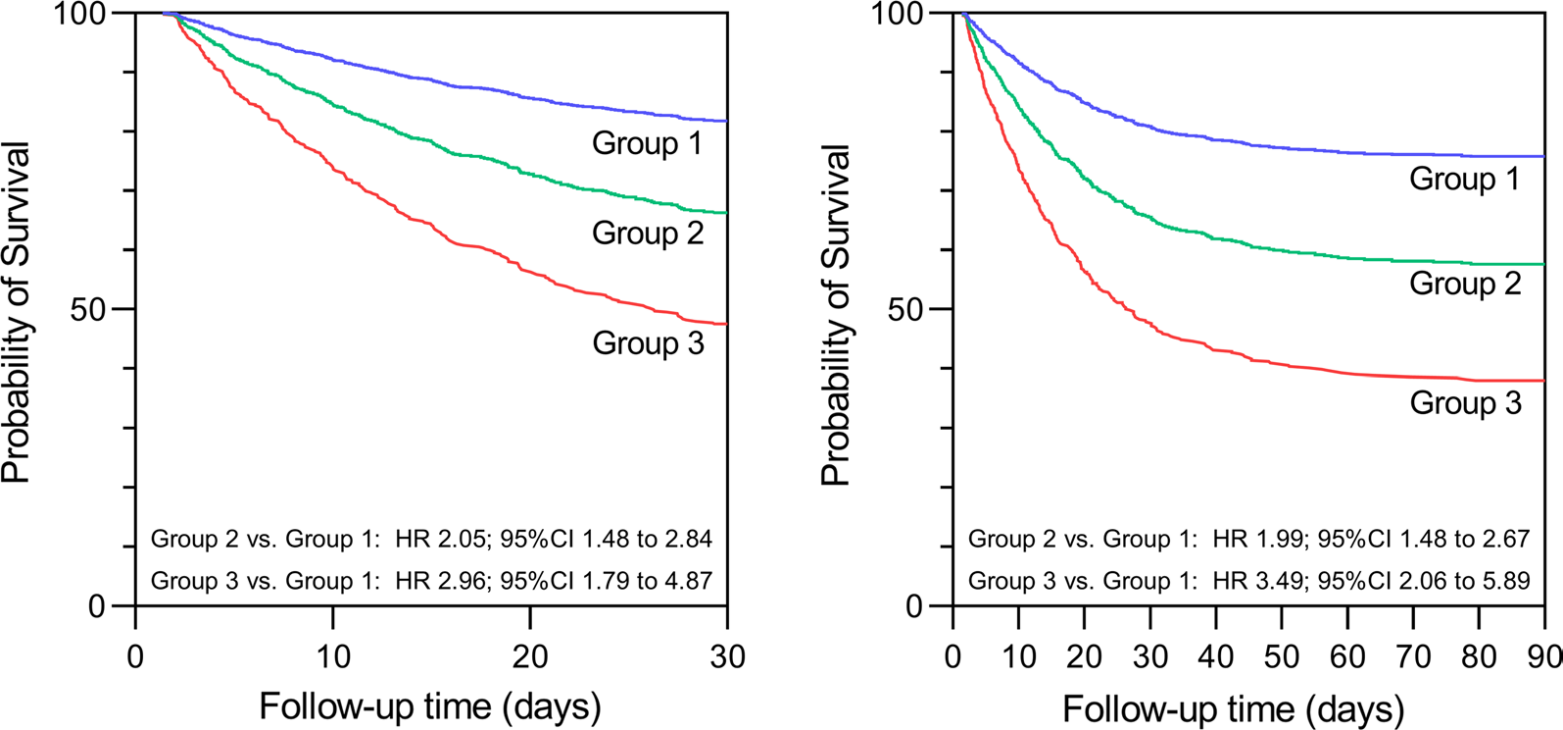
Survival analysis by GBTM lactate groups. Multivariable Cox proportional hazard analyses by GBTM lactate groups with respect to 30- (left) and 90-day mortality (right). CI, confidence intervals; HR, hazard ratio

**Fig. 4:**
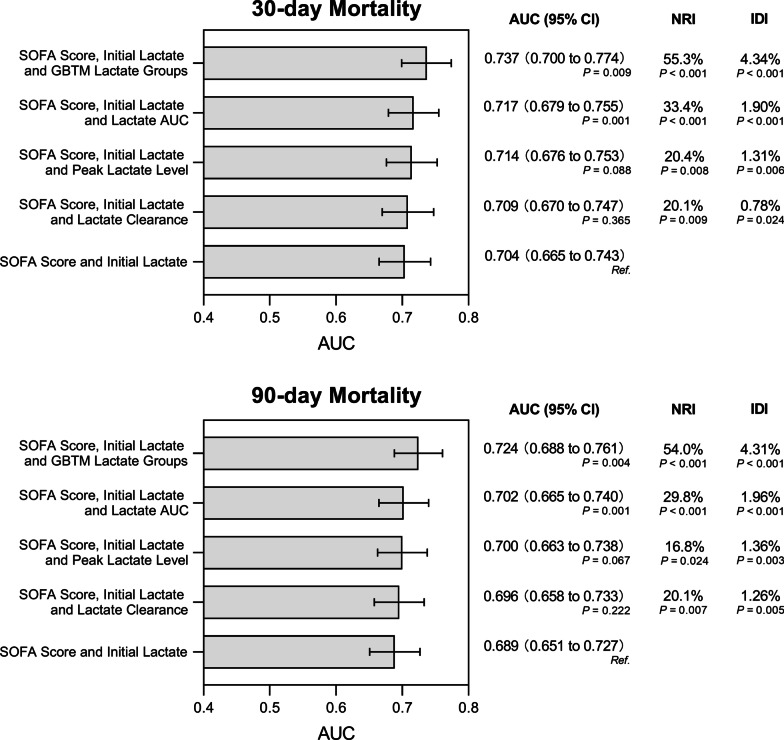
Comparison of diagnostic capacity. The AUC, IDI, and NRI for the combined assessment of diagnostic capacity when incorporating the GBTM lactate groups, peak lactate level, lactate clearance and lactate AUC into the established model (SOFA score + initial lactate). All *P* values represent comparisons with the established model (SOFA score + initial lactate). AUC, area under curve; IDI, integrated discrimination improvement; GBTM, group-based trajectory modeling; NRI, net reclassification improvement; SOFA score, Sequential Organ Failure Assessment score

The GBTM lactate group 3 exhibited the worst (HR for 30-day mortality: 2.96, 95% CI 1.79–4.87, *P* < 0.001; HR for 90-day mortality: 3.49, 95% CI 2.06–5.89, *P* < 0.001) outcomes followed by the GBTM lactate group 2 (HR for 30-day mortality: 2.05, 95% CI 1.48–2.84, *P* < 0.001; HR for 90-day mortality: HR: 1.99, 95% CI 1.48–2.67, *P* < 0.001). The Cox hazard curves are shown in Fig. 3. When the GBTM lactate groups were added to the initial lactate models + SOFA score, there were significant increases in the established models' AUC value for short- (0.737 vs. 0.704, *P* = 0.009) and long-term outcomes (0.724 vs. 0.689, *P* = 0.004) with enhanced risk of reclassification capacity (positive and significant continuous NRI values), as well as IDI values. Furthermore, the GBTM lactate groups + initial lactate + SOFA score yielded the highest predictive performance compared with the models where the lactate AUC, lactate clearance and peak lactate level were added to the initial lactate + SOFA score (Fig. 4). These results were consistent in initial lactate + APACHE II score models (Additional file 1: Fig. S3).

### Comparison of the nonlinearity on age, eGFR, SOFA score, and APACHE II score between GBTM lactate groups and initial lactate groups assignment with respect to short- and long-term outcomes

The baseline characteristics based on initial lactate levels are shown in Additional file 1: Table S7. The initial lactate groups 1, 2, and 3 accounted for 54.6% (n = 415), 27.2% (n = 207) and 18.2% (n = 138) of the cases, respectively. Compared with initial lactate group 1, group 2 experienced a 1.62- (HR: 1.62, 95% CI 1.19–2.22) and 1.77-fold (HR: 1.77, 95% CI 1.34–2.36) greater risk of short- and long-term mortality, respectively. The short- (HR: 1.80, 95% CI 1.25–2.59) and long-term (HR: 1.70, 95% CI 1.20–2.40) risks in group 3 were indistinguishable from those in group 2 (Additional file 1: Fig. S4).

**Fig. 5:**
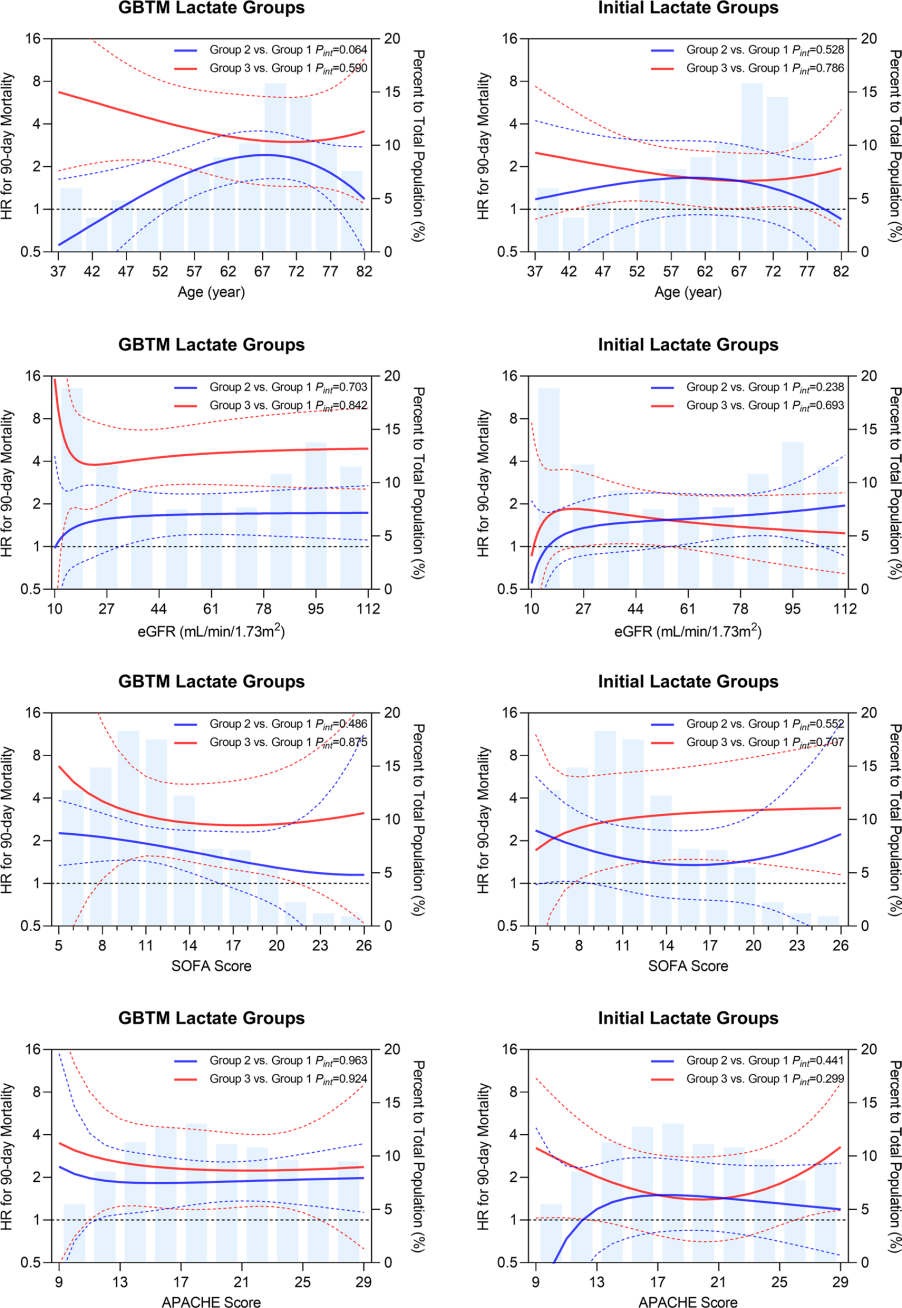
MFPI analysis. A plot of estimated HRs of the nonlinearity on age, eGFR, SOFA score, and APACHE II score between GBTM lactate groups and initial lactate groups assignment with respect to 90-day mortality. The solid red and blue lines represent the estimated HRs of groups 3 and 2 compared to group 1. The red and blue dashed lines near them represent the CIs corresponding to the HRs, respectively. The horizontal black dashed lines denote HR of 1. A transparent blue histogram of the background represents the distribution of continuous variables. Except for SOFA scores, for the rest three continuous indicators, we adopted the 10th to the 90th quantile of distribution. APACHE II score, Acute Physiology And Chronic Health Evaluation II score; CI, confidence intervals; eGFR, estimated glomerular filtration rate; GBTM, group-based trajectory modeling; HR, hazard ratio; SOFA score, Sequential Organ Failure Assessment score

Using the MFPI procedure, non-linearities in survival were found for the GBTM lactate groups/initial lactate groups combined with age, eGFR, SOFA score, and APACHE II score. As seen in Fig. 5, GBTM lactate group 3 experienced higher 90-day mortality than group 2 and was clearly separated and consistent across multiple continuous indicators (despite the overlap of CI). However, based on the initial lactate groups, the risk of groups 3 and 2 could not be well discriminated. GBTM lactate groups can better identify high-risk patients on four continuous scales. Compared with the GBTM lactate group 1, age, eGFR, SOFA score (8–21) and APACHE II score (11–26) were statistically significant in 100%, 100%, 72.7% (40/55), and 60.0% (33/55) of GBTM lactate group 3 patients [with the lower 95% CI above the line of equivalence (HR: 1)]. In the GBTM lactate group 2, the age, eGFR, SOFA score and APACHE II achieved statistical significance in 61.1% (181/296), 65.9% (195/296), 72.6% (215/296), and 87.8% (260/296) of the total population in group 2. In contrast, no significant difference in the four continuous parameters were found in the initial lactate groups. Hence, high-risk patients could not be identified. No significant interaction was observed across the four continuous parameters. The 30-day mortality was also roughly consistent with the above results (Additional file 1: Fig. S5).

## Discussion

In this retrospective cohort study of ARDS patients complicated with sepsis in the ICU, we validated the presence of elevated lactate levels in ARDS patients with sepsis. Importantly, this is the first study to document the value of GBTM-based serial blood lactate evaluations to significantly improve the diagnostic capacity of traditional critical care evaluation systems compared to previously described lactate approaches. Interestingly, we used the MFPI algorithm to demonstrate that GBTM lactate groups yield superior performance in identifying high-risk patients than initial lactate groups for both short- and long-term outcomes.

Sepsis/multiple organ failure is well-established as a common cause of mortality in ARDS patients, and improvement in clinical outcomes may depend more on the management of sepsis/multiple organ failure than on oxygenation measures alone [20]. It is widely acknowledged that blood lactate level is a useful marker of the severity of sepsis or multiple organ failure, with higher levels predictive of higher mortality [9]. The peak lactate level has been initially applied in various blood lactate evaluation systems [8]. However, it should be borne in mind that tissue hypoperfusion and hypoxia may be present with elevated lactate levels before routine hemodynamic measurements change [30]. Therefore, initial measurements of lactate levels in the ICU are valuable for early identification. Mikkelsen et al. found that the initial serum lactate was associated with mortality independent of clinically apparent organ dysfunction and shock in patients admitted to the emergency department with severe sepsis [10]. Early initial lactate-guided therapy can significantly reduce ICU mortality [11]. Overwhelming evidence substantiates that initial blood lactate levels ranging from 2.0 to 4.0 mmol/L are associated with poor outcomes [9, 10, 12, 13]. The initial lactate is simple to calculate and readily available in all institutions. However, a single measurement of the lactate concentration can be challenging to interpret, especially in patients with ARDS, since it reflects a balance between lactate production and elimination. In this study, we divided the initial lactate into three groups with 2.0 mmol/L and 4.0 mmol/L as cut-off values for comparison with the GBTM lactate groups. The high initial lactate group exhibited lower mortality rates than the corresponding GBTM-based lactate group. The initial lactate groups exhibited lower survival during survival curve analysis than the GBTM lactate groups (Additional file 1: Fig. S4). More importantly, we specifically compared GBTM lactate groups with the initial lactate groups using the MFPI algorithm. Both high (group 3) and low (group 2) initial lactate groups did not correspond well to relative high-risk and low-risk clinical outcomes on the pre-specified continuous age, eGFR, SOFA score, and APACHE II score, nor could it obtain the high-risk interval with statistical significance. Taken together, our findings substantiate that GBTM lactate groups are more advantageous than the initial lactate groups.

Since continuous blood lactate levels assessment may provide more information than individual values, serial blood lactate measurements have been proposed [31]. Subsequently, the concepts of hyperlactatemia duration [15], lactate clearance [17], and lactate AUC [16] have been recommended. In our study, GBTM-based lactate groups could roughly cover patients with high initial blood lactate, peak lactate level and lactate AUC values (Additional file 1: Fig. S2B**)**, with group 3 more tightly associated with the three parameters than group 2 or 1. Accordingly, the GBTM groups could account for findings in the above four lactate evaluation systems. Findings in GBTM lactate groups were not driven by individual parameters such as initial lactate, peak lactate value, lactate AUC, etc., but by a combination of these. In addition, GBTM lactate groups exhibited significantly enhanced diagnostic performance of the established model of SOFA score/APACHE II score with the addition of initial lactate. Meanwhile, the AUC, NRI and IDI obtained by adding GBTM lactate groups were the largest compared with established models where lactate AUC, lactate clearance and the peak lactate values were added, suggesting better diagnostic efficacy.

Several limitations and shortcomings were present in this study. Given its retrospective design, our study may have been subject to systematic errors and biases with missing values for serial lactate measurements. Nevertheless, the random forest data interpolation method based on machine learning [27], known to outperform all other data interpolation methods [32], was used to handle the missing data. Moreover, as an observational study, we could not establish a causal relationship between lactate groups and clinical outcomes and exclude the impact of potential confounders. Indeed, we could not include these potential confounders during multivariate analysis due to the lack of data, such as body mass index. Given the strong association between lactate groups and short- or long-term outcomes and the consistency of trends for continuous indicators during MFPI, it is highly unlikely that these factors can affect the conclusions of this study. Due to a lack of information (such as bilateral infiltration on chest roentgenogram), we could not use the Berlin definition [33] to define patients with ARDS. However, we exclude patients with clinical entities can present with PaO_2_/FiO_2_ < 300 mmHg without being ARDS (atelectasis, volume overload, left heart failure) based on the initial diagnosis at ICU admission. Moreover, due to the study's retrospective nature, we could not determine the causal relationship between ARDS and sepsis. Given that the sample size in the study was small, our findings may have been biased in favor of effect size inflation, and the statistical ability may be insufficient to detect the correct number of tracks when using GBTM. Hence, future GBTM-based prospective studies with larger sample sizes are required to validate our findings.

## Conclusions

The GBTM-based serial lactate evaluation method significantly improved the diagnostic capacity of traditional critical care evaluation systems and possessed many advantages over previously described lactate evaluation systems, especially over groups based on initial lactate levels. Importantly, GBTM-based serial lactate evaluation improved the identification of individuals at high risk of short- and long-term mortality in our retrospective cohort study of ARDS patients complicated with sepsis in the ICU.

## Supplementary Information

**Additional file 1: Table S1**. Definition of study variables. **Table S2**. Missing rates of study variables. **Table S3**. Initial ICU admission diagnosis of eligible patients. **Table S4**. Baseline characteristics by study outcomes. **Table S5**. The parameter for fitted models with different numbers of latent groups and different degrees of polynomials. **Table S6**. Baseline lactate characteristics between GBTM lactate groups. **Table S7**. Baseline characteristics by initial lactate. **Figure S1**. Calculation of lactate AUC. **Figure S2**. Comparisons and descriptions of GBTM lactate groups by initial lactate, peak lactate level, lactate clearance and lactate AUC. **Figure S3**. The ROC with associated AUC, IDI, and NRI, for the combined assessment of diagnostic capacity when incorporating the GBTM lactate groups, peak lactate level, lactate clearance and lactate AUC respectively into the established model (APACHE II score + initial lactate). **Figure S4**. Multivariable Cox proportional hazard analyses by initial lactate groups with respect to 30- (left) and 90-day mortality (right). **Figure S5**. A plot of estimated HRs of the nonlinearity on continuous age, eGFR, SOFA score, and APACHE II score between GBTM lactate groups and initial lactate groups assignment with respect to 30-day mortality.

## Data Availability

The datasets used and/or analyzed during the current study are available from the corresponding author on reasonable request.

## Abbreviations

AIC: Akaike information criterion
ALT: Alanine aminotransferase
APACHE II: Acute Physiology And Chronic Health Evaluation II
ARDS: Acute respiratory distress syndrome
AST: Aspartate aminotransferase
AUC: Area under the curve
BIC: Bayesian information criterion
CI: Confidence interval
CRRT: Continuous renal replacement therapy
DBP: Diastolic blood pressure
eGFR: Estimated glomerular filtration rate
GBTM: Group-based trajectory modeling
GCS: Glasgow Coma Scale
HR: Hazard ratio
ICU: Intensive care units
IDI: Integrated discrimination improvement
MFPI: Multivariable fractional polynomial interaction
NRI: Net reclassification improvement
RBC: Red blood cell
SBP: Systolic blood pressure
STROBE: Strengthening the Reporting of Observational Studies in Epidemiology
SOFA: Sequential Organ Failure Assessment
WBC: White blood cell
