# Supplementary material for: Risk stratification of patients with acute respiratory distress syndrome complicated with sepsis using lactate trajectories
Source: BMC Pulm Med. 2022 Sep 7;22:339. doi: 10.1186/s12890-022-02132-6 (PMC9451114; doi:10.1186/s12890-022-02132-6)
Supplement: Supplementary file 1 — Additional file 1: Table S1. Definition of study variables. Table S2. Missing rates of study variables. Table S3. Initial ICU admission diagnosis of eligible patients. Table S4. Baseline characteristics by study outcomes. Table S5. The parameter for fitted models with different numbers of latent groups and different degrees of polynomials. Table S6. Baseline lactate characteristics between GBTM lactate groups. Table S7. Baseline characteristics by initial lactate. Figure S1. Calculation of lactate AUC. Figure S2. Comparisons and descriptions of GBTM lactate groups by initial lactate, peak lactate level, lactate clearance and lactate AUC. Figure S3. The ROC with associated AUC, IDI, and NRI, for the combined assessment of diagnostic capacity when incorporating the GBTM lactate groups, peak lactate level, lactate clearance and lactate AUC respectively into the established model (APACHE II score + initial lactate). Figure S4. Multivariable Cox proportional hazard analyses by initial lactate groups with respect to 30- (left) and 90-day mortality (right). Figure S5. A plot of estimated HRs of the nonlinearity on continuous age, eGFR, SOFA score, and APACHE II score between GBTM lactate groups and initial lactate groups assignment with respect to 30-day mortality. [file 12890_2022_2132_MOESM1_ESM.docx]

**Additional file 1**

***Risk Stratification of Patients with Acute Respiratory Distress Syndrome Complicated with Sepsis Using Lactate Trajectories***

Content of Additional file 1

Additional file 1: Table

**Additional file 1: Table S1.** Definition of study variables.

**Additional file 1: Table S2.** Missing rates of study variables.

**Additional file 1: Table S3.** Initial ICU admission diagnosis of eligible patients.

**Additional file 1: Table S4.** Baseline characteristics by study outcomes.

**Additional file 1: Table S5.** The parameter for fitted models with different numbers of latent groups and different degrees of polynomials.

**Additional file 1: Table S6.** Baseline lactate characteristics between GBTM lactate groups.

**Additional file 1: Table S7.** Baseline characteristics by initial lactate.

Additional file 1: Figure

**Additional file 1: Figure S1.** Calculation of lactate AUC.

**Additional file 1: Figure S2.** Comparisons and descriptions of GBTM lactate groups by initial lactate, peak lactate level, lactate clearance and lactate AUC.

**Additional file 1: Figure S3.** The ROC with associated AUC, IDI, and NRI, for the combined assessment of diagnostic capacity when incorporating the GBTM lactate groups, peak lactate level, lactate clearance and lactate AUC respectively into the established model (APACHE II score + initial lactate).

**Additional file 1: Figure S4.** Multivariable Cox proportional hazard analyses by initial lactate groups with respect to 30- (left) and 90-day mortality (right).

**Additional file 1: Figure S5.** A plot of estimated HRs of the nonlinearity on continuous age, eGFR, SOFA score, and APACHE II score between GBTM lactate groups and initial lactate groups assignment with respect to 30-day mortality.

**Additional file 1: Table S1.** Definition of study variables.

| Variables | Definition | Continuous/Categorical/Binary |
| --- | --- | --- |
| Demographics | | |
| Age | Years after birth | Continuous |
| Sex | Female and Male | Binary, Female/Male |
| Previous History |  |  |
| Hypertension | Self-reported use of medications to control blood pressure or previously measured SBP ≥140mmHg or DBP ≥90mmHg | Binary, Yes/No |
| Diabetes | Self-reported use of medications/lifestyle intervention to control blood glucose or physician-diagnosed type 2 diabetes mellitus | Binary, Yes/No |
| CAD | Self-reported history of CAD | Binary, Yes/No |
| Stroke | Self-reported history of stroke, include hemorrhagic stroke and ischemic stroke | Binary, Yes/No |
| Renal failure | Self-reported history of renal failure | Binary, Yes/No |
| Cancer | Self-reported history of Cancer | Binary, Yes/No |
| On-admission clinical characteristics | | |
| SBP | SBP measured on admission | Continuous, in mmHg |
| DBP | DBP measured on admission | Continuous, in mmHg |
| Heart rate | Heart rate measured on admission | Continuous, in beats per minute |
| eGFR | eGFR on admission calculated by CKD-EPI formula | Continuous, in mL/min/1.73m^2^ |
| Fluid intake | Hourly intake within 24 hours of admission, including oral intake and intravenous intake, etc | Continuous, in mL/hour |
| Fluid outtake | Hourly output within 24 hours of admission, including urine volume, ultrafiltration (if CRRT), and drainage, etc | Continuous, in mL/hour |
| RBC | Red blood cell count measured on admission by routine blood test | Continuous, in 10^9^/L |
| WBC | White blood cell count measured on admission by routine blood test | Continuous, in 10^9^/L |
| Platelet | Platelet count measured on admission by routine blood test | Continuous, in 10^9^/L |
| Hemoglobin | Hemoglobin measured on admission by routine blood test | Continuous, in g/L |
| ALT | Plasma alanine aminotransferase measured on admission | Continuous, in U/L |
| AST | Plasma aspartate aminotransferase measured on admission | Continuous, in U/L |
| Albumin | Plasma albumin measured on admission | Continuous, in g/L |
| Oxygenation index | The ratio of PaO_2_ to FiO_2_ was obtained by arterial blood gas analysis on admission | Continuous, in mmHg |
| GCS score | GCS score evaluated on admission | Continuous |
| APACHE II Score | APACHE II score evaluated on admission | Continuous |
| SOFA Score | SOFA score evaluated on admission | Continuous |
| Emergency surgical operation | Surgical procedures in the first 24 h of admission | Binary, Yes/No |
| Invasive mechanical ventilation | Invasive mechanical ventilation during the entire ICU stays | Binary, Yes/No |
| Other in-hospital treatment within 24 hours of admission | | |
| Antibiotics | Antibiotics use within 24h after admission | Binary, Yes/No |
| Sedative and analgesic | Sedative and analgesic use within 24h after admission (including: propofol, dexmedetomidine, midazolam, opioids etc.) | Binary, Yes/No |
| Glucocorticoids | Glucocorticoids use within 24h after admission | Binary, Yes/No |
| CRRT | CRRT use within 24h after admission | Binary, Yes/No |
| LMWH | LMWH use within 24h after admission | Binary, Yes/No |
| Sodium Bicarbonate | Sodium Bicarbonate use within 24h after admission | Binary, Yes/No |
| Vasoactive medications | Vasoactive medications (norepinephrine/dopamine) use within 24h after admission | Binary, Yes/No |
| Blood component transfusions | Blood component transfusions (red cells, platelets, plasma, cryoprecipitated AHF, and granulocytes) use within 24h after admission | Binary, Yes/No |

**Abbreviations:** ALT, alanine aminotransferase; APACHE II, Acute Physiology And Chronic Health Evaluation Ⅱ; AST, aspartate aminotransferase; CKD-EPI, Chronic Kidney Disease Epidemiology Collaboration; CRRT, continuous renal replacement therapy, DBP, diastolic blood pressure; eGFR, estimated glomerular filtration rate; GCS, Glasgow Coma Scale; LMWH, low molecular weight heparin; RBC, red blood cell; SBP, systolic blood pressure; SOFA, Sequential Organ Failure Assessment; WBC, white blood cell.

**Additional file 1: Table S2.** Missing rates of study variables.

| Variables | N (%)  n=760 |
| --- | --- |
| AaDO_2_ | 66 (8.68) |
| Sodium | 2 (0.26) |
| Potassium | 1 (0.13) |
| Initial lactate | 11 (1.44) |
| Lactate at 6h after admission | 63 (8.29) |
| Lactate at 12h after admission | 76 (10.0) |
| Lactate at 18h after admission | 68 (8.95) |
| Lactate at 24h after admission | 48 (6.32) |
| Lactate at 30h after admission | 77 (10.1) |
| Lactate at 36h after admission | 98 (12.9) |
| Lactate at 42h after admission | 74 (9.74) |
| Lactate at 48h after admission | 46 (6.05) |
| Fluid intake | 37 (4.87) |
| Fluid outtake | 37 (4.87) |

**Abbreviations:** AaDO_2_, alveolar-arterial oxygen difference.

**Additional file 1: Table S3.** Initial ICU admission diagnosis of eligible patients.

| Admission diagnosis | N (%)  n=760 |
| --- | --- |
| Pneumonia | 302 (39.7) |
| Cancer | 66 (8.68) |
| Stroke | 54 (7.11) |
| Infection (intra-abdominal) | 44 (5.79) |
| Renal dysfunction* | 59 (7.76) |
| Trauma | 39 (5.13) |
| Acute pancreatitis | 23 (3.03) |
| Infection (CNS) | 22 (2.89) |
| Hypohepatia | 20 (2.63) |
| Infection (soft tissue) | 19 (2.50) |
| Toxic chemicals | 16 (2.11) |
| Infection (urinary tract) | 12 (1.58) |
| DKA | 11 (1.45) |
| Infection (E.N.T) | 7 (0.92) |
| PE | 6 (0.79) |
| Infection (others)† | 5 (0.66) |
| Others | 55 (7.24) |

This table shows the initial diagnosis at the time of admission to the ICU of eligible patients, which may reflect the cause of ARDS or sepsis. *Renal dysfunction does not include patients with fluid overload or heart failure due to renal dysfunction. †Other infections refer to infections outside the CNS, E.N.T, intra-abdominal and urinary tract.

**Abbreviations:** ARDS, acute respiratory distress syndrome; CNS, central nervous system; DKA,

diabetic ketoacidosis; E.N.T, earnosethroat; ICU, intensive care unit; PE, pulmonary embolism.

**Additional file 1: Table S4.** Baseline characteristics by study outcomes.

| Characteristics | Total n=760 | 30-day mortality | | *P* value | 90-day mortality | | *P* value |
| --- | --- | --- | --- | --- | --- | --- | --- |
|  |  | 30-day mortality (+) n=256 | 30-day mortality (-) n=504 |  | 90-day mortality (+) n=303 | 90-day mortality (-) n=457 |  |
| Lactate levels within 48 hours of admission, mmol/L | | | | | | | |
| Initial lactate | 1.80 (1.30 to 3.30) | 2.50 (1.50 to 4.55) | 1.60 (1.20 to 2.70) | <0.001 | 2.40 (1.50 to 4.30) | 1.60 (1.20 to 2.40) | <0.001 |
| Lactate at 6h after admission | 1.90 (1.30 to 2.90) | 2.53 (1.60 to 4.40) | 1.70 (1.20 to 2.50) | <0.001 | 2.40 (1.60 to 4.00) | 1.70 (1.20 to 2.47) | <0.001 |
| Lactate at 12h after admission | 1.80 (1.30 to 2.70) | 2.40 (1.60 to 3.60) | 1.60 (1.20 to 2.30) | <0.001 | 2.40 (1.55 to 3.00) | 1.60 (1.20 to 2.30) | <0.001 |
| Lactate at 18h after admission | 1.70 (1.30 to 2.50) | 2.20 (1.60 to 3.10) | 1.60 (1.20 to 2.10) | <0.001 | 2.08 (1.50 to 3.00) | 1.50 (1.20 to 2.00) | <0.001 |
| Lactate at 24h after admission | 1.61 (1.30 to 2.30) | 2.10 (1.50 to 3.00) | 1.50 (1.20 to 2.00) | <0.001 | 2.05 (1.40 to 2.80) | 1.50 (1.20 to 2.00) | <0.001 |
| Lactate at 30h after admission | 1.60 (1.20 to 2.20) | 2.00 (1.40 to 2.85) | 1.50 (1.20 to 1.94) | <0.001 | 1.90 (1.40 to 2.70) | 1.46 (1.20 to 1.90) | <0.001 |
| Lactate at 36h after admission | 1.60 (1.20 to 2.10) | 1.90 (1.40 to 2.62) | 1.42 (1.15 to 1.90) | <0.001 | 1.90 (1.40 to 2.60) | 1.40 (1.10 to 1.80) | <0.001 |
| Lactate at 42h after admission | 1.51 (1.20 to 2.20) | 1.90 (1.40 to 2.60) | 1.40 (1.10 to 1.90) | <0.001 | 1.82 (1.40 to 2.60) | 1.40 (1.10 to 1.80) | <0.001 |
| Lactate at 48h after admission | 1.50 (1.20 to 2.10) | 1.90 (1.40 to 2.60) | 2.40 (1.10 to 1.89) | <0.001 | 1.90 (1.30 to 2.50) | 1.40 (1.10 to 1.80) | <0.001 |
| Peak lactate level within 48 hours after admission, mmol/L | | | | | | | |
|  | 2.70 (1.90 to 4.60) | 3.70 (2.50 to 6.20) | 2.50 (1.80 to 3.90) | <0.001 | 3.60 (2.41 to 6.10) | 2.30 (1.71 to 3.60) | <0.001 |
| Lactate clearance within 48 hours after admission, % | | | | | | | |
|  | 20.0 (-16.7 to 46.7) | 24.4 (-16.7 to 48.6) | 17.4 (-16.8 to 48.1) | 0.850 | 25.0 (-18.1 to 51.3) | 15.4 (-15.4 to 44.4) | 0.250 |
| Lactate AUC within 48 hours of admission | | | | | | | |
|  | 0.08 (0.06 to 0.11) | 0.10 (0.07 to 0.14) | 0.07 (0.06 to 0.10) | <0.001 | 0.99 (0.07 to 0.13) | 0.07 (0.57 to 0.09) | <0.001 |
| Demographics |  |  |  |  |  |  |  |
| Age, year | 62.2±16.7 | 66.1±14.1 | 60.2±17.6 | <0.001 | 66.2±14.2 | 59.6±17.8 | <0.001 |
| Male, n (%) | 464 (61.1) | 153 (59.8) | 311 (61.7) | 0.640 | 186 (61.4) | 278(60.8) | 0.940 |
| Previous history |  |  |  |  |  |  |  |
| Hypertension, n (%) | 401 (52.8) | 145 (56.6) | 265 (50.8) | 0.140 | 172 (56.8) | 229 (50.1) | 0.0750 |
| Diabetes, n (%) | 235 (30.9) | 80 (31.3) | 155 (30.8) | 0.930 | 93 (30.7) | 142 (31.1) | 0.940 |
| CAD, n (%) | 174 (22.9) | 63 (24.6) | 111 (22.0) | 0.470 | 77 (25.4) | 97 (21.2) | 0.190 |
| Stroke, n (%) | 167 (22.0) | 56 (21.9) | 111 (22.0) | 1.000 | 73 (24.1) | 94 (20.6) | 0.280 |
| Renal failure, n (%) | 76 (10.0) | 17 (6.60) | 59 (11.7) | 0.030 | 23 (7.60) | 53 (11.6) | 0.084 |
| Cancer, n (%) | 95 (12.5) | 41 (16.0) | 54 (10.7) | 0.048 | 46 (15.2) | 49 (10.7) | 0.074 |
| On-admission clinical characteristics | | | | | | | |
| SBP, mmHg | 137.3±35.1 | 131.5±35.4 | 140.3±34.7 | 0.001 | 132.3±35.9 | 140.7±34.2 | 0.001 |
| DBP, mmHg | 70.3±19.1 | 66.3±18.6 | 72.4±19.0 | <0.001 | 66.5±18.8 | 72.9±18.9 | <0.001 |
| Heart rate, bpm | 101.6±24.1 | 103.7±25.5 | 100.6±23.3 | 0.097 | 103.5±24.8 | 100.4±23.4 | 0.082 |
| eGFR, mL/min/1.73m^2^ | 58.8±41.7 | 54.8±37.3 | 60.8±43.7 | 0.061 | 55.1±37.6 | 61.2±44.0 | 0.050 |
| Fluid intake, mL/hour | 161.9 (113.3 to 243.9) | 170.7 (120.5 to 265.2) | 156.7 (109.5 to 230.0) | 0.040 | 165.2 (120.0 to 263.7) | 157.5 (109.4 to 229.4) | 0.054 |
| Fluid outtake, mL/hour | 136.0 (84.0 to 243.9) | 133.2 (84.1 to 231.8) | 138.9 (83.8 to 235.5) | 0.570 | 129.0 (84.5 to 234.2) | 140.0 (83.6 to 232.9) | 0.520 |
| RBC, 10^9^/L | 3.36 (2.63 to 4.12) | 3.16 (2.52 to 3.93) | 3.48 (2.73 to 4.17) | <0.001 | 3.16 (2.51 to 3.91) | 3.51 (2.80 to 4.22) | <0.001 |
| WBC, 10^9^/L | 10.8 (7.70 to 16.0) | 11.6 (7.73 to 16.91) | 10.5 (7.69 to 15.3) | 0.110 | 11.2 (7.53 to 16.8) | 10.6 (7.77 to 15.4) | 0.400 |
| Platelet, 10^9^/L | 165.0 (92.0 to 230.0) | 147.0 (78.0 to 207.0) | 172.0 (98.5 to 238.0) | 0.001 | 151.0 (80.0 to 214.0) | 172.0 (99.0 to 237.0) | 0.009 |
| Hemoglobin, g/L | 101.0 (80.0 to 124.5) | 94.5 (76.0 to 120.1) | 104.0 (83.0 to 126.5) | 0.008 | 93.0 (76.0 to 120.0) | 105.0 (85.0 to 128.0) | <0.001 |
| ALT, U/L | 36.0 (22.0 to 77.0) | 40.0 (23.0 to 87.0) | 34.0 (21.0 to 69.0) | 0.071 | 40.0 (23.0 to 87.0) | 33.0 (21.0 to 67.0) | 0.010 |
| AST, U/L | 53.0 (32.0 to 116.0) | 64.0 (37.0 to 138.0) | 49.0 (30.0 to 105.5) | <0.001 | 65.0 (37.0 to 166.0) | 46.0 (30.0 to 101.0) | <0.001 |
| Albumin, g/L | 30.0 (26.0 to 34.0) | 30.0 (25.0 to 33.0) | 31.0 (27.0 to 34.0) | 0.001 | 30.0 (25.0 to 33.0) | 31.0 (27.0 to 35.0) | <0.001 |
| GCS Score | 13 (7 to 15) | 10 (5 to 15) | 14 (8 to 15) | <0.001 | 10 (5 to 15) | 14 (8 to 15) | <0.001 |
| APACHE II Score | 18 (13 to 24) | 22 (16 to 27) | 17 (12 to 22) | <0.001 | 21 (16 to 27) | 17 (12 to 22) | <0.001 |
| SOFA Score | 11 (8 to 14) | 13 (10 to 17) | 10 (8 to 13) | <0.001 | 12 (10 to 17) | 10 (8 to 13) | <0.001 |
| PaO_2_/FiO_2_, mmHg | | | | <0.001 |  |  | <0.001 |
| < 100 | 307 (40.4) | 76 (29.7) | 227 (44.7) |  | 91 (30.0) | 216 (47.3) |  |
| 100 ~ 200 | 323 (42.5) | 114 (44.5) | 213 (41.9) |  | 139 (45.9) | 184 (40.3) |  |
| > 300 | 130 (17.1) | 66 (25.8) | 68 (13.4) |  | 73 (24.1) | 57 (12.5) |  |
| Emergency surgical operation, n (%) | | | | | | | |
|  | 93 (12.2) | 57 (22.3) | 36 (7.10) | <0.001 | 64 (21.1) | 29 (6.30) | <0.001 |
| Invasive mechanical ventilation, n (%) | | | | | | | |
|  | 482 (63.4) | 229 (89.5) | 253 (50.2) | <0.001 | 265 (87.5) | 217 (47.5) | <0.001 |
| Other in-hospital treatment within 24 hours of admission | | | | | | | |
| Antibiotics, n (%) | 676 (88.9) | 233 (91.0) | 443 (50.2) | 0.220 | 277 (91.4) | 399 (87.3) | 0.098 |
| Sedative and analgesic, n (%) | 476 (88.9) | 174 (68.0) | 298 (59.1) | 0.018 | 204 (67.3) | 268 (58.6) | 0.018 |
| Glucocorticoid, n (%) | 353 (46.4) | 142 (55.5) | 211 (41.9) | <0.001 | 161 (53.1) | 192 (42.0) | 0.003 |
| CRRT, n (%) | 260 (34.2) | 90 (35.2) | 170 (33.7) | 0.750 | 107 (35.3) | 153 (35.5) | 0.640 |
| LWMH, n (%) | 99 (13.0) | 30 (11.7) | 69 (13.7) | 0.490 | 35 (11.6) | 64 (14.0) | 0.380 |
| Sodium bicarbonate, n (%) | 358 (47.1) | 133 (52.0) | 225 (44.6) | 0.065 | 159 (52.5) | 199 (43.5) | 0.018 |
| Vasoactive medications, n (%) | 245 (32.2) | 124 (48.4) | 121 (24.0) | <0.001 | 138 (45.5) | 107 (23.4) | <0.001 |
| Blood component transfusion, n (%) | 274 (36.1) | 122 (47.7) | 152 (30.2) | <0.001 | 146 (48.2) | 128 (28.0) | <0.001 |

**Abbreviations:** ALT, alanine aminotransferase; APACHE II, Acute Physiology And Chronic Health Evaluation Ⅱ; AST, aspartate aminotransferase; AUC, area under curve; CRRT, continuous renal replacement therapy, DBP, diastolic blood pressure; eGFR, estimated glomerular filtration rate; GCS, Glasgow Coma Scale; ICU, intensive care unit; LMWH, low molecular weight heparin; RBC, red blood cell; SBP, systolic blood pressure; SOFA, Sequential Organ Failure Assessment; WBC, white blood cell.

**Additional file 1: Table S5.** The parameter for fitted models with different numbers of latent groups and different degrees of polynomials.

| Polynomial | N | Percentage | Average Posterior Probabilities | Odds of Correct Classification by Weighted Posterior Proportions | Expection Based on the Posterior Probabilities | BIC | AIC |
| --- | --- | --- | --- | --- | --- | --- | --- |
| Intercept (0) | 568 | 74.73% | 98.37% | 20.48683 | 74.54% | -4954.67 | -4945.40 |
| Intercept (0) | 192 | 25.26% | 95.95% | 70.16305 | 25.45% |  |  |
| Intercept (0) | 547 | 71.97% | 98.37% | 23.66644 | 71.85% | -4836.40 | -4824.82 |
| Linear (1) | 213 | 28.02% | 96.23% | 65.244 | 28.14% |  |  |
| Linear (1) | 576 | 76.31% | 98.78% | 24.60727 | 76.70% | -4902.93 | -4891.34 |
| Intercept (0) | 186 | 23.68% | 94.41% | 55.6451 | 23.29% |  |  |
| Linear (1) | 558 | 73.42% | 98.45% | 23.19857 | 73.28% | -4798.24 | -4784.34 |
| Linear (1) | 202 | 26.57% | 96.23% | 70.04358 | 26.71% |  |  |
| Intercept (0) | 547 | 71.97% | 98.31% | 22.9923 | 71.75% | -4836.09 | -4822.19 |
| Quadratic (2) | 213 | 28.02% | 96.44% | 68.93543 | 28.24% |  |  |
| Linear (1) | 557 | 72.31% | 98.44% | 24.071 | 72.42% | -4798.51 | -4782.29 |
| Quadratic (2) | 203 | 27.69% | 95.55% | 56.35752 | 27.58% |  |  |
| Quadratic (2) | 551 | 73.28% | 98.47% | 23.7234 | 73.19% | -4801.44 | -4782.91 |
| Quadratic (2) | 211 | 26.71% | 96.17% | 68.71783 | 26.80% |  |  |
| Quadratic (2) | 580 | 76.31% | 98.82% | 25.45845 | 76.9% | -4905.41 | -4979.26 |
| Intercept (0) | 180 | 23.68% | 94.19% | 53.72975 | 23.20% |  |  |
| Quadratic (2) | 558 | 73.42% | 98.48% | 23.59749 | 73.33% | -4801.09 | -4784.87 |
| Linear (1) | 202 | 26.57% | 96.14% | 68.54208 | 26.66% |  |  |
| Intercept (0) | 410 | 53.94% | 95.91% | 20.09954 | 53.87% | -4317.69 | -4303.79 |
| Intercept (0) | 297 | 39.07% | 94.15% | 25.00669 | 39.16% |  |  |
| Intercept (0) | 53 | 6.97% | 97.61% | 547.1045 | 6.96% |  |  |
| Linear (1) | 427 | 56.18% | 96.23% | 20.0289 | 56.07% | -4290.88 | -4274.66 |
| Intercept (0) | 282 | 37.11% | 94.05% | 26.81918 | 37.11% |  |  |
| Intercept (0) | 51 | 6.71% | 98.51% | 907.1207 | 6.81% |  |  |
| Intercept (0) | 405 | 53.28% | 96.90% | 27.13157 | 53.53% | -4121.63 | -4105.42 |
| Linear (1) | 301 | 39.60% | 94.91% | 28.68717 | 39.42% |  |  |
| Intercept (0) | 54 | 7.10% | 97.41% | 498.3649 | 7.03% |  |  |
| Intercept (0) | 408 | 53.68% | 95.66% | 19.25142 | 53.37% | -4303.91 | -4287.69 |
| Intercept (0) | 296 | 38.94% | 94.59% | 26.91521 | 39.40% |  |  |
| Linear (1) | 56 | 7.36% | 96.86% | 397.4235 | 7.21% |  |  |
| Linear (1) | 412 | 54.21% | 96.89% | 25.95804 | 54.60% | -4110.87 | -4092.34 |
| Linear (1) | 296 | 38.94% | 94.13% | 25.70832 | 38.45% |  |  |
| Intercept (0) | 52 | 6.84% | 98.47% | 865.2239 | 6.94% |  |  |
| Linear (1) | 423 | 55.65% | 96.18% | 20.16062 | 55.56% | -4277.53 | -4259.00 |
| Intercept (0) | 285 | 37.5% | 93.80% | 25.37204 | 37.36% |  |  |
| Linear (1) | 52 | 6.84% | 99.04% | 1368.671 | 7.07% |  |  |
| Intercept (0) | 386 | 53.15% | 96.87% | 27.05504 | 53.36% | -4110.36 | -4091.82 |
| Linear (1) | 322 | 39.60% | 95.06% | 29.49592 | 39.52% |  |  |
| Linear (1) | 54 | 7.23% | 97.14% | 443.9151 | 7.11% |  |  |
| Linear (1) | 409 | 53.81% | 97.07% | 27.7791 | 54.39% | -4099.66 | -4078.81 |
| Linear (1) | 297 | 39.07% | 94.17% | 27.7791 | 38.56% |  |  |
| Linear (1) | 54 | 7.10% | 97.32% | 480.4188 | 7.03% |  |  |
| Quadratic (2) | 429 | 56.44% | 96.15% | 19.53489 | 56.16% | -4293.26 | -4274.73 |
| Intercept (0) | 280 | 36.84% | 94.33% | 28.33506 | 37.02% |  |  |
| Intercept (0) | 51 | 6.71% | 98.47% | 883.6876 | 6.80% |  |  |
| Intercept (0) | 403 | 53.02% | 97.05% | 28.71122 | 53.44% | -4118.35 | -4099.81 |
| Quadratic (2) | 303 | 39.86% | 94.73% | 27.57129 | 39.47% |  |  |
| Intercept (0) | 54 | 7.10% | 97.93% | 623.7782 | 7.07% |  |  |
| Intercept (0) | 408 | 53.68% | 95.60% | 19.03351 | 53.32% | -4306.80 | -4288.27 |
| Intercept (0) | 296 | 38.94% | 94.63% | 27.09137 | 39.43% |  |  |
| Quadratic (2) | 56 | 7.36% | 97.11% | 430.4533 | 7.24% |  |  |
| Quadratic (2) | 409 | 53.81% | 97.10% | 28.00282 | 54.46% | -4111.30 | -4088.13 |
| Quadratic (2) | 297 | 39.07% | 94.11% | 25.51772 | 38.53% |  |  |
| Intercept (0) | 54 | 7.10% | 97.26% | 472.1731 | 6.99% |  |  |
| Quadratic (2) | 424 | 55.78% | 96.18% | 20.11628 | 55.62% | -4282.89 | -4259.73 |
| Intercept (0) | 282 | 37.10% | 94.21% | 27.40015 | 37.28% |  |  |
| Quadratic (2) | 54 | 7.10% | 97.25% | 464.9221 | 7.08% |  |  |
| Intercept (0) | 403 | 53.02% | 96.87% | 27.2704 | 53.23% | -4109.49 | -4086.32 |
| Quadratic (2) | 302 | 39.73% | 95.08% | 29.54082 | 39.57% |  |  |
| Quadratic (2) | 55 | 7.23% | 98.06% | 656.2476 | 7.18% |  |  |
| Quadratic (2) | 409 | 53.81% | 96.88% | 26.25969 | 54.19% | -4102.56 | -4074.76 |
| Quadratic (2) | 296 | 38.94% | 94.53% | 27.43017 | 38.67% |  |  |
| Quadratic (2) | 55 | 7.23% | 97.52% | 514.0325 | 7.12% |  |  |
| Quadratic (2) | 410 | 53.94% | 96.99% | 27.01765 | 54.44% | -4102.72 | -4079.56 |
| Linear (1) | 296 | 38.94% | 94.26% | 26.20609 | 38.52% |  |  |
| Linear (1) | 54 | 7.10% | 97.28% | 474.3676 | 7.02% |  |  |
| Linear (1) | 409 | 53.81% | 96.88% | 26.26401 | 54.20% | -4096.64 | -4073.48 |
| Quadratic (2) | 296 | 38.94% | 94.51% | 27.3061 | 38.68% |  |  |
| Linear (1) | 55 | 7.23% | 97.22% | 457.9579 | 7.10% |  |  |
| Linear (1) | 409 | 53.81% | 97.02% | 27.40407 | 54.34% | -4102.45 | -4079.28 |
| Linear (1) | 297 | 39.07% | 94.22% | 25.94725 | 38.58% |  |  |
| Quadratic (2) | 54 | 7.10% | 97.65% | 548.2532 | 7.06% |  |  |
| Quadratic (2) | 409 | 53.81% | 96.91% | 26.50872 | 54.23% | -4099.86 | -4074.37 |
| Quadratic (2) | 296 | 38.94% | 94.48% | 27.17435 | 38.66% |  |  |
| Linear (1) | 55 | 7.23% | 97.19% | 453.4381 | 7.10% |  |  |
| Quadratic (2) | 409 | 53.81% | 97.06% | 27.73017 | 54.39% | -4105.51 | -4080.03 |
| Linear (1) | 297 | 39.07% | 94.15% | 25.70623 | 38.54% |  |  |
| Quadratic (2) | 54 | 7.10% | 97.62% | 541.382 | 7.06% |  |  |
| Linear (1) | 409 | 53.81% | 96.85% | 26.02285 | 54.16% | -4099.34 | -4073.86 |
| Quadratic (2) | 296 | 38.94% | 94.56% | 27.55964 | 38.70% |  |  |
| Quadratic (2) | 55 | 7.23% | 97.55% | 519.0016 | 7.13% |  |  |
| Quadratic (2) | 409 | 53.81% | 96.88% | 26.25952 | 54.20% | -4102.56 | -4074.76 |
| Quadratic (2) | 296 | 38.94% | 94.54% | 27.43036 | 38.68% |  |  |
| Quadratic (2) | 55 | 7.23% | 97.53% | 514.0338 | 7.13% |  |  |

The grey shading parameter is finally selected (we decided on a model with three latent groups and linear, quadratic, and linear polynomials, respectively).

**Additional file 1: Table S6.** Baseline lactate characteristics between GBTM lactate groups.

| Characteristics | Total n=760 | GBTM lactate groups | | | *P* value |
| --- | --- | --- | --- | --- | --- |
|  |  | Group 1 n=409 | Group 2 n=296 | Group 3 n=55 |  |
| Lactate levels within 48 hours of admission, mmol/L | | | | | |
| Initial lactate | 1.80 (1.30 to 3.30) | 1.30 (1.10 to 1.80) | 3.00 (2.10 to 3.71) | 6.60 (4.30 to 10.7) | <0.001 |
| Lactate at 6h after admission | 1.90 (1.30 to 2.90) | 1.40 (1.10 to 1.70) | 2.70 (2.10 to 3.70) | 7.40 (5.20 to 11.3) | <0.001 |
| Lactate at 12h after admission | 1.80 (1.30 to 2.70) | 1.30 (1.10 to 1.60) | 2.56 (2.10 to 3.20) | 6.50 (4.70 to 8.90) | <0.001 |
| Lactate at 18h after admission | 1.70 (1.30 to 2.50) | 1.40 (1.10 to 1.60) | 2.30 (1.80 to 2.90) | 6.20 (4.60 to 9.20) | <0.001 |
| Lactate at 24h after admission | 1.61 (1.30 to 2.30) | 1.30 (1.10 to 1.60) | 2.20 (1.80 to 2.65) | 6.10 (4.30 to 8.20) | <0.001 |
| Lactate at 30h after admission | 1.60 (1.20 to 2.20) | 1.30 (1.10 to 1.60) | 2.10 (1.70 to 2.60) | 5.60 (4.30 to 8.50) | <0.001 |
| Lactate at 36h after admission | 1.60 (1.20 to 2.10) | 1.30 (1.10 to 1.55) | 2.00 (1.70 to 2.36) | 5.30 (3.90 to 6.50) | <0.001 |
| Lactate at 42h after admission | 1.51 (1.20 to 2.20) | 1.30 (1.10 to 1.52) | 1.93 (1.52 to 2.40) | 4.90 (3.80 to 6.90) | <0.001 |
| Lactate at 48h after admission | 1.50 (1.20 to 2.10) | 1.20 (1.00 to 1.50) | 1.90 (1.50 to 2.30) | 4.30 (3.20 to 7.20) | <0.001 |
| Peak lactate level within 48 hours after admission, mmol/L | | | | | |
|  | 2.70 (1.90 to 4.60) | 1.90 (1.60 to 2.50) | 4.10 (3.10 to 5.60) | 11.2 (7.40 to 15.9) | <0.001 |
| Lactate clearance within 48 hours after admission | | | | | |
|  | 20.0 (-16.7 to 46.6) | 6.67 (-21.4 to 33.3) | 35.7 (-3.95 to 60.6) | 30.4 (-33.3 to 61.0) | <0.001 |
| Lactate AUC within 48 hours of admission | | | | | |
|  | 0.08 (0.06 to 0.11) | 0.06 (0.05 to 0.07) | 0.11 (0.09 to 0.13) | 0.26 (0.22 to 0.39) | <0.001 |

**Abbreviations:** AUC, area under curve; GBTM, group-based trajectory modeling.

**Additional file 1: Table S7.** Baseline characteristics by initial lactate

| Characteristics | Total n=760 | Initial Lactate Groups | | | *P* value |
| --- | --- | --- | --- | --- | --- |
|  |  | Group 1 n=415 | Group 2 n=207 | Group 3 n=138 |  |
| Demographics |  |  |  |  |  |
| Age, year | 62.2±16.7 | 61.6±17.8 | 64.2±15.8 | 60.9±14.7 | 0.130 |
| Male, n (%) | 464 (61.1) | 253(61.0) | 127 (61.4) | 84 (60.9) | 1.000 |
| Previous history |  |  |  |  |  |
| Hypertension, n (%) | 401 (52.8) | 225 (54.2) | 109 (52.7) | 67 (48.6) | 0.520 |
| Diabetes, n (%) | 235 (30.9) | 139 (33.5) | 63 (30.4) | 33 (23.9) | 0.110 |
| CAD, n (%) | 174 (22.9) | 104 (25.1) | 42 (20.3) | 28 (20.3) | 0.310 |
| Stroke, n (%) | 167 (22.0) | 95 (22.9) | 51 (24.6) | 21 (15.2) | 0.087 |
| Renal failure, n (%) | 76 (10.0) | 57 (13.7) | 9 (4.30) | 10 (7.20) | <0.001 |
| Cancer, n (%) | 95 (12.5) | 41 (9.90) | 30 (14.5) | 24 (17.4) | 0.039 |
| On-admission clinical characteristics | | | | | |
| SBP, mmHg | 137.3±35.1 | 145.7±34.7 | 130.1±31.6 | 123.0±34.4 | <0.001 |
| DBP, mmHg | 70.3±19.1 | 73.5±18.9 | 68.2±18.1 | 64.0±19.0 | <0.001 |
| Heart rate, bpm | 101.6±24.1 | 96.4±22.2 | 105.9±25.2 | 111.0±24.1 | <0.001 |
| eGFR, mL/min/1.73m^2^ | 58.8±41.7 | 57.6±44.0 | 64.8±39.5 | 53.3±36.5 | 0.029 |
| Fluid intake, mL/hour | 161.9 (113.3 to 243.9) | 147.8 (103.0 to 221.8) | 164.4 (121.3 to 229.1) | 214.2 (144.5 to 317.2) | <0.001 |
| Fluid outtake, mL/hour | 136.0 (84.0 to 243.9) | 141.2 (85.7 to 235.5) | 122.8 (81.3 to 202.7) | 144.8 (85.6 to 249.2) | 0.160 |
| RBC, 10^9^/L | 3.36 (2.63 to 4.12) | 3.31 (2.60 to 4.03) | 3.48 (2.86 to 4.17) | 3.25 (2.55 to 4.23) | 0.079 |
| WBC, 10^9^/L | 10.8 (7.70 to 16.0) | 10.1 (7.25 to 13.8) | 12.3 (8.77 to 18.8) | 13.1 (8.54 to 19.2) | <0.001 |
| Platelet, 10^9^/L | 165.0 (92.0 to 230.0) | 172.0 (112.0 to 237.0) | 160.0 (78.0 to 231.0) | 135.5 (65.0 to 195.0) | <0.001 |
| Hemoglobin, g/L | 101.0 (80.0 to 124.5) | 99.0 (78.0 to 122.0) | 106.0 (83.0 to 130.0) | 96.0 (78.0 to 126.0) | 0.065 |
| ALT, U/L | 36.0 (22.0 to 77.0) | 30.0 (19.0 to 63.0) | 38.0 (22.0 to 84.0) | 47.5 (29.0 to 170.0) | <0.001 |
| AST, U/L | 53.0 (32.0 to 116.0) | 43.0 (28.0 to 78.0) | 60.0 (36.0 to 165.0) | 104.5 (53.0 to 374.0) | <0.001 |
| Albumin, g/L | 30.0 (26.0 to 34.0) | 31.0 (27.0 to 34.0) | 30.0 (25.0 to 34.0) | 28.5 (25.0 to 33.0) | 0.006 |
| GCS Score | 13 (7 to 15) | 14 (8 to 15) | 11 (5 to 15) | 10 (4 to 15) | <0.001 |
| APACHE II Score | 18 (13 to 24) | 17 (12 to 22) | 20 (15 to 25) | 21 (16 to 29) | <0.001 |
| SOFA Score | 11 (8 to 14) | 10 (8 to 12) | 12 (9 to 15) | 14 (11 to 18) | <0.001 |
| PaO_2_/FiO_2_, mmHg |  |  |  |  | <0.001 |
| < 100 | 307 (40.4) | 192 (46.3) | 76 (36.7) | 39 (28.3) |  |
| 100 ~ 200 | 323 (42.5) | 178 (42.9) | 87 (42.0) | 58 (42.0) |  |
| > 300 | 130 (17.1) | 45 (10.8) | 44 (21.3) | 41 (29.7) |  |
| Emergency surgical operation, n (%) | | | | | |
|  | 93 (12.2) | 35 (8.40) | 33 (15.9) | 25 (18.1) | 0.002 |
| Invasive mechanical ventilation, n (%) | | | | | |
|  | 482 (63.4) | 217 (52.3) | 148 (71.5) | 117 (84.8) | <0.001 |
| Other in-hospital treatment within 24 hours of admission | | | | | |
| Antibiotics, n (%) | 676 (88.9) | 357 (86.0) | 194 (93.7) | 125 (90.6) | 0.011 |
| Sedative and analgesic, n (%) | 476 (88.9) | 242 (58.3) | 131 (63.3) | 99 (71.7) | 0.016 |
| Glucocorticoid, n (%) | 353 (46.4) | 157 (37.8) | 110 (53.1) | 86 (62.3) | <0.001 |
| CRRT, n (%) | 260 (34.2) | 148 (35.7) | 50 (24.2) | 62 (44.9) | <0.001 |
| LWMH, n (%) | 99 (13.0) | 60 (14.5) | 25 (12.1) | 14 (10.1) | 0.410 |
| Sodium bicarbonate, n (%) | 358 (47.1) | 172 (41.4) | 95 (45.9) | 91 (65.9) | <0.001 |
| Vasoactive medications, n (%) | 245 (32.2) | 82 (19.8) | 88 (42.5) | 75 (54.3) | <0.001 |
| Blood component transfusion, n (%) | 274 (36.1) | 131 (31.6) | 71 (34.3) | 72 (52.2) | <0.001 |

**Abbreviations:** ALT, alanine aminotransferase; APACHE II, Acute Physiology And Chronic Health Evaluation Ⅱ; AST, aspartate aminotransferase; AUC, area under curve; CRRT, continuous renal replacement therapy, DBP, diastolic blood pressure; eGFR, estimated glomerular filtration rate; GCS, Glasgow Coma Scale; LMWH, low molecular weight heparin; RBC, red blood cell; SBP, systolic blood pressure; SOFA, Sequential Organ Failure Assessment; WBC, white blood cell.


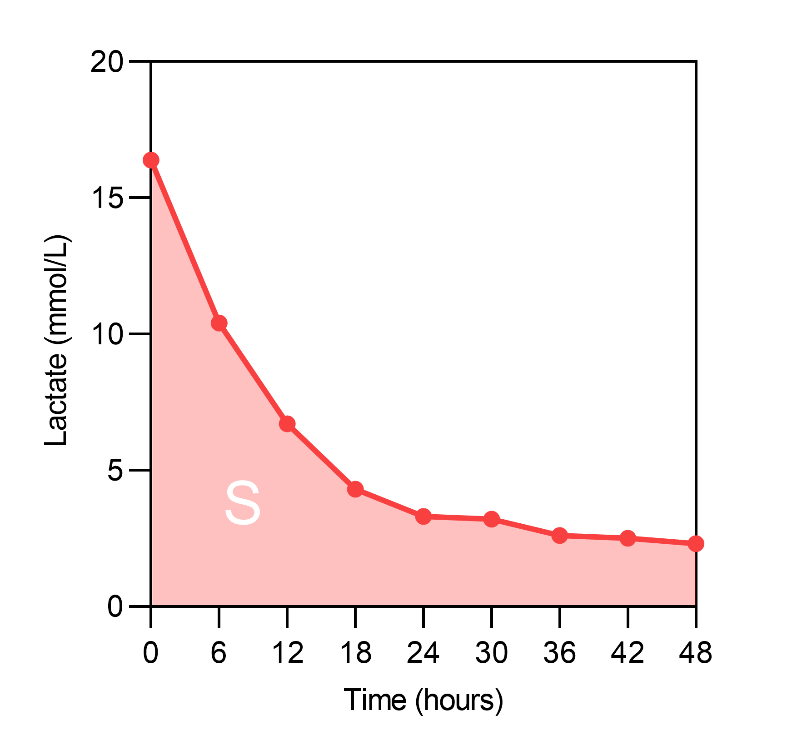


**Additional file 1: Figure S1.** Calculation of lactate AUC.

Lactate AUC=S/(20×48); the upper limit for measuring lactate in arterial blood gas is 20mmol/L.

**Abbreviations:** AUC, area under curve.

**
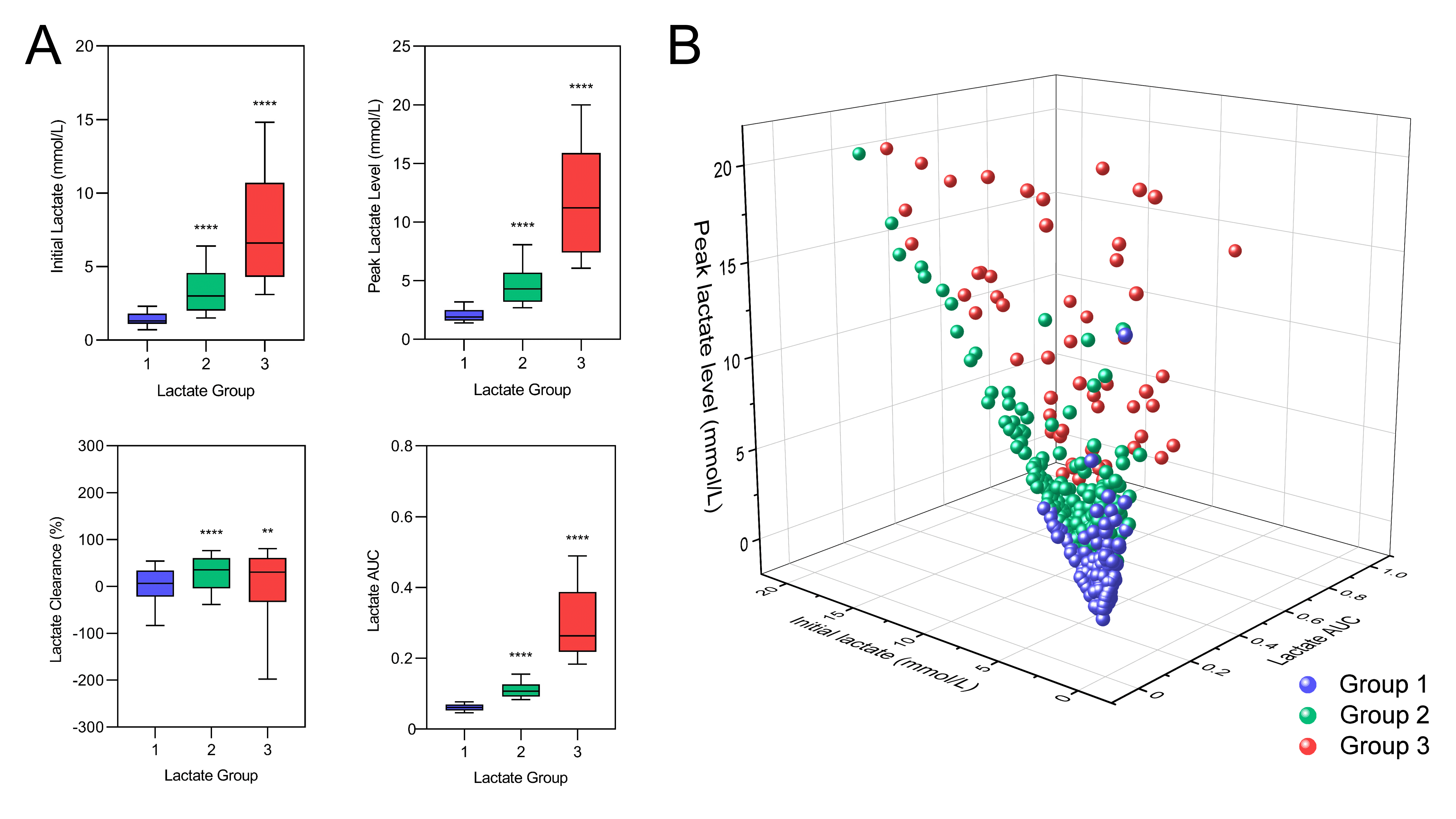
**

**Additional file 1: Figure S2.** Comparisons and descriptions of GBTM lactate groups by initial lactate, peak lactate level, lactate clearance and lactate AUC.

**A.** Comparisons of GBTM lactate groups by initial lactate, peak lactate level, lactate clearance and lactate AUC, presented with Box-Whisker plots, in which the boxes extend from the 25^th^ percentile to the 75^th^ percentile, with a line at the median. The whiskers show the 10^th^ percentile to the 90^th^ percentile. Statistical comparisons were performed with Mann-Whitney U test, and GBTM lactate group 1 was used as reference. ns indicates nonsignificant. *Indicates *P*<0.05. **Indicates *P*<0.01. ***Indicates *P*<0.001. ****Indicates *P*<0.0001.

**B.** The 3D scatter plots of GBTM lactate groups are described in three dimensions: initial lactate, peak lactate level, and lactate AUC.

**Abbreviations:** AUC, area under curve; GBTM, group-based trajectory modeling.





**Additional file 1: Figure S3.** The ROC with associated AUC, IDI, and NRI, for the combined assessment of diagnostic capacity when incorporating the GBTM lactate groups, peak lactate level, lactate clearance and lactate AUC respectively into the established model (APACHE II score + initial lactate).

All *P*'s represent comparisons with established model (APACHE II score + initial lactate).

**Abbreviations:** APACHE II score, Acute Physiology And Chronic Health Evaluation Ⅱ score; AUC, area under curve; IDI, integrated discrimination improvement; GBTM, group-based trajectory modeling; NRI, net reclassification improvement.





**Additional file 1: Figure S4.** Multivariable Cox proportional hazard analyses by initial lactate groups with respect to 30- (left) and 90-day mortality (right).

Abbreviations: CI, confidence intervals; HR, hazard ratio.

**

**

**Additional file 1: Figure S5.** A plot of estimated HRs of the nonlinearity on continuous age, eGFR, SOFA score, and APACHE II score between GBTM lactate groups and initial lactate groups assignment with respect to 30-day mortality.

The solid red and blue lines represent the estimated HRs of the group 3 and 2 compared to the group 1, respectively, and the red and blue dashed lines near them represent the CIs corresponding to the HRs, respectively. The horizontal black dashed lines denote HR of 1. A transparent blue histogram of the background represents the distribution of continuous variables. Except for SOFA scores, For the rest three continuous indicators, we adopted the 10^th^ to the 90^th^ quantile of distribution.

**Abbreviations:** APACHE II score, Acute Physiology And Chronic Health Evaluation Ⅱ score; CI, confidence intervals; eGFR, estimated glomerular filtration rate; GBTM, group-based trajectory modeling; HR, hazard ratio; SOFA score, Sequential Organ Failure Assessment score.
